# Utility of serum indices in a particular case of serum protein electrophoresis

**DOI:** 10.11613/BM.2022.030802

**Published:** 2022-08-05

**Authors:** Massimo Daves, Andrea Piccin, Cristina Vicidomini, Antonia De Luisi, Andrea Mega

**Affiliations:** 1 Clinical Biochemical Laboratory, Hospital of Bolzano, Bolzano, Italy; 2 Northern Ireland Blood Transfusion Service, Belfast, UK; 3 Department of Internal Medicine V, Medical University of Innsbruck, Innsbruck, Austria; 4 Department of Industrial Engineering, University of Trento, Trento, Italy; 5 Division of Gastroenterology, Hospital of Bolzano, Bolzano, Italy

**Keywords:** capillary zone electrophoresis, interferences, serum indices

## Abstract

Screening and measurement of monoclonal (M) proteins are commonly performed using capillary zone electrophoresis (CZE). The identification of M-protein or monoclonal component (CM) is an essential requirement for diagnosis and monitoring of monoclonal gammopathies. The detection of CM has been largely improved by CZE. Capillary electrophoresis estimates CM more accurately, because absence of variation due to different dye binding affinities of proteins as instead seen with agarose gel electrophoresis. However, interferences can be present in CZE. This occurs because all substances absorbing at 200 nm can be identified. Recognition and handling of specimens exhibiting such interferences is essential to ensure accurate diagnostic and patient safety. We herein report on an unusual case of serum protein electrophoresis, to highlight that laboratory staff must be aware of and familiarise with the information provided by laboratory instruments. For example, in the case of serum indices, about specimen quality.

## Introduction

At the present, the best test available to detect the presence of M-protein or monoclonal component (CM) is the protein electrophoresis (in agarose gel or with capillary method) (*1*). The identification of CM is an essential requirement for diagnosis and monitoring of monoclonal gammopathies. Electrophoresis, as well as immunofixation, can be carried out in serum and urine (*2*). Capillary zone electrophoresis (CZE) is used for screening and quantification of CM. The detection of any CM must be very accurate because the associated clinical and therapeutic implications (*3*). Capillary electrophoresis estimates CM more accurately, because absence of variation due to different dye binding affinities of proteins, which is found in agarose gel electrophoresis. However, interferences can be present in CZE. This occurs because all substances absorbing at 200 nm can be detected (*4*). Therefore, exogenous substances such as iodinated contrast agents, may absorb UV light at similar wavelengths and may affect protein detection in CZE (*5*). The most commonly seen interferences are: i) irregularities in the beta-1 and alpha-2 globulin fraction due to haemolysis; ii) the identification of the cathodic shoulder on the albumin side (*e.g.* massive hyperlipidaemia); iii) irregularities due to hyperbilirubinemia (*4*).

Interference due to haemolysis (H), jaundice (I) and lipids (L) cause very often preanalytical errors. Prompt recognition and expert handling of such interferences is essential to ensure data accuracy and patient safety (*6*). From some years the analytical systems based on spectrophotometry can analyse serum indices (SI). We herein discuss an unusual case of serum protein electrophoresis. Laboratory and medical staff must be aware of and familiarise with these kind of information. For example, in the case of serum indices, about specimen quality.

## Case report

We report on the case of a 50-year-old woman with a history of advanced liver cirrhosis following an autoimmune hepatitis. The patient underwent 6 monthly blood tests prescribed by her general practitioner. The examinations included complete blood cell count (CBC), routine coagulation study (prothrombin time and activated partial thromboplastin time), and biochemistry study (creatinine, bilirubin, total protein, alfa-fetoprotein, liver transaminases, gamma glutamyl-transferase, alkaline phosphatase, and serum protein electrophoresis). The CBC (determined with the XN 9000 haematology analyser, Sysmex, Kobe, Japan) show a mild anaemia (haemoglobin (Hb) 111 g/L) with reduced mean corpuscular volume (MCV) (75 fL, reference Interval (RI) 80-96 fL), reduced mean corpuscular haemoglobin (MCH) (23.6 pg, RI 27-31) and an increased red cell distribution width (RDW) (20.9%, RI 11.5-14.5). The white blood cell (WBC), red blood cell (RBC), platelet (PLT) and differential WBC count were within their respective reference interval. The routine coagulation study (performed with ACL_Top, Instrumentation Laboratory, Milano, Italy) showed only a slight prolongation of the activated partial thromboplastin time (1.26 Ratio, RI 0.7-1.20) and a normal value of prothrombin time. The results of the biochemical parameters measured (using Cobas e, Roche Diagnostics GmbH, Mannheim, Germany) are reported in Table 1.

**Table 1:** Results of the biochemical parameters measured and results from capillary zone electrophoresis.

**Parameter (unit)**	**Result**	**Reference interval**
Creatinine (µmol/L)	62	44-88
GGT (U/L)	440	< 40
AST (U/L)	131	< 35
ALT (U/L)	164	< 35
ALP (U/L)	667	35-130
Total Bilirubin (µmol/L)	126	< 24
Direct Bilirubin (µmol/L)	125	< 5
CHE (U/L)	4268	4500-12,000
Alfa-fetoprotein (kIU/L)	3.7	< 4.8
Total protein (g/L)	75	66-83
Albumin (%)	56	55-68
Albumin (g/L)	43.9	38.0-47.5
Alfa-1 (%)	4.4	1.5-5.0
Alfa-1 (g/L)	3.4	2.0-3.5
Alfa-2 (%)	10	6-12
Alfa-2 (g/L)	8.1	5.0-8.5
Beta-1 (%)	6	< 8
Beta-1 (g/L)	5	4-7
Beta-2 (%)	6	< 8
Beta-2 (g/L)	4.5	1.0-4.5
Gamma (%)	18	11-21
Gamma (g/L)	14.2	0.8-1.4
GGT - gamma-glutamyl transferase. CHE – Cholinesterase. ALT - Alanine aminotransferase. AST - Aspartate aminotransferase. ALP - Alkaline phosphatase.		

**Figure 1:**
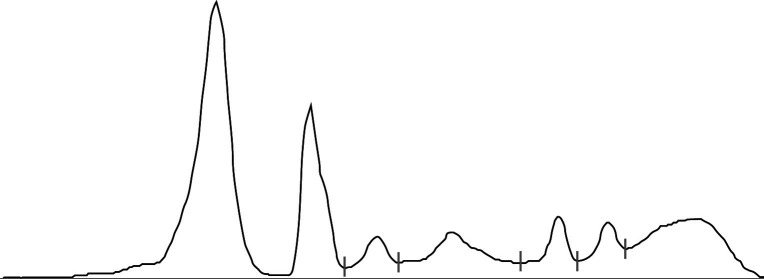
Capillary zone electrophoresis electropherogram.

Serum protein electrophoresis was performed using Capillarys 2 Flex-Piercing (Sebia, Lisses, France). The CZE electropherogram showed an atypical pattern with the presence of a peak in the cathode area with respect to albumin (Figure 1). Serum sample aspect was unremarkable on visual inspection, no clear alterations were detected. Moreover, an immunofixation (Hydragel, SEBIA France) ruled out the presence of a CM.

The patient signed an informed consent form for anonymous publication of medical data.

To exclude an abnormal increase in alpha-fetoprotein (Elecsys AFP, Cobas e, Roche Diagnostics GmbH) with a possible underestimation due to hook effect, the sample was retested after several dilutions. Nevertheless, the same finding was confirmed.

**Figure 2:**
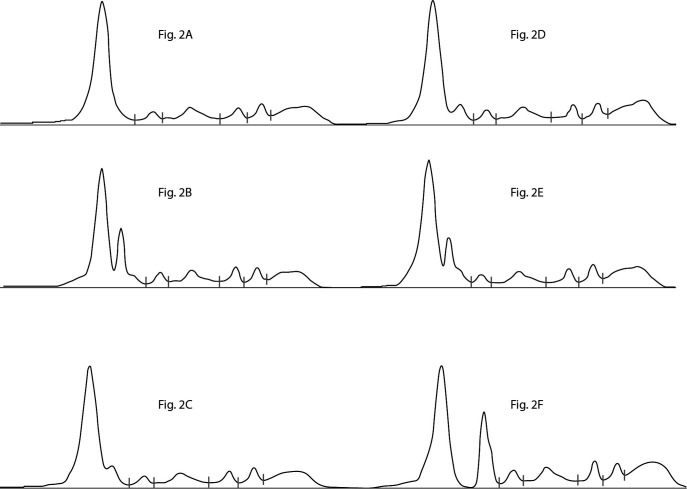
Previous protein electrophoresis performed. **(A)** Protein electrophoresis (July 2019). Total cholesterol 8.91 mmol/L, total bilirubin 67 µmol/L. Lipemic index was not yet available. **(B)** (October 2019) Total cholesterol 18.79 mmol/L, bilirubin not measured. **(C)** (December 2019) Total cholesterol 11.03 mmol/L, bilirubin 58 µmol/L. **(D)** (November 2020) bilirubin 64 µmol/L, cholesterol non measured, lipemic index 17. **(E)** (March 2021) bilirubin 128 µmol/L, lipemic index 27, cholesterol not measured. **(F)** August 2021 bilirubin 126 µmol/L, total cholesterol 25.4 mmol/L, lipemic index 38.

We contacted the attending physician who dismissed the use of any contrast media or drugs. We repeated the electrophoresis on a fresh sample after a few days. Once again we found the atypical pattern as previously observed. However, on this occasion testing was carried out by using nephelometry (Atellica NEPH630 Siemens, Marburg, Germany). This was necessary to exclude abnormal increases, alpha 1 antitrypsin (AAT) and alpha 1 acid glycoprotein (AAG) which were found to be only slightly increased (AAT: 3.84 g/L; RI <2 and AAG: 1.76 g/L, RI <1.2). The albumin measured with the nephelometer was found to be 38 g/L (RI 35-53 g/L). We also measured apoliprotein A and apoliprotein B which respectively gave the following values: 0.718 g/L (RI 1.10-2.15) and 2.68 g/L (RI 0.55-1.40). To investigate a case of bis-albuminemia we extracted from the Laboratory Computer System the electrophoresis performed in previous years (see Figure 2). The presence of congenital bis-albuminemia appeared unlikely given the previous electrophoretic traces that not always show the anomalous peak and while sometimes was present but to a different extent.

Looking at the electrophoresis previously performed, we noted an electrophoresis performed two years earlier with an abnormal peak placed between albumin and the alpha 1 zone. Interestingly, a marked hypercholesterolemia (18.79 mmol/L) was also reported (Figure 2B). Therefore, we analysed in detail the other available reports. Some common biochemical alterations such as the elevation of the bilirubin concentration with values always between about 51 and 120 μmol/L were seen. However, the bilirubin concentrations did not have a correlation with the abnormal peak detected.

Conversely a direct linear relationship with total cholesterol values and/or with the lipemic index was identified. Interestingly the lipemic index performed on the Roche analyzer in conjunction with the last biochemical tests performed was 38. This was a significant increased in value despite the normal appearance of the serum. We therefore measured the total cholesterol (Cholesterol Gen.2 Cobas C, Roche Diagnostics GmbH) also for the relationship previously observed between lipemic index values and cholesterol with the appearance of the abnormal peak which resulted 25.4 mmol/L (desirable value < 5.17 mmol/L). In Figure 2 the electrophoretic tracings performed previously with the possibly available values of bilirubin, cholesterol and/or lipemic index are illustrated. In this case, the increased lipemic index (despite the normal appearance of the serum) allowed us to suspect an abnormal concentration of lipids and lipoproteins responsible for the abnormal peak. The presence of hypercholesterolemia was therefore confirmed with the direct measurement of total cholesterol except for the finding of only slightly increased triglyceride values (2.15 mmol/L, RI 0.34-1.69) measured on the Cobas c 702.

## Discussion

Several scientific studies invoke the need for objective systems for the detection of the serum indices (SI), discouraging the manual methods linked to the observation of the sample by the operator with consequent subjectivity and poor reproducibility. Furthermore the automated analysis of the SI does not lead to increases significant costs and not even significant lengthening of the turnaround time (TAT) (*7*). Another very interesting aspect is the use of SI for ruling out the presence of important pathologies. Consider, for example, the potential role of the lipemia index in the evaluation of lipemic alteration metabolisms, as in our case. Consider also the possible economic savings due to the careful use of these indices which in various cases could avoid to run unnecessary tests or on the contrary could increase the diagnostic effectiveness by unmasking conditions in which a more in-depth evaluation is necessary (*4*, *8*, *9*, *11*). Another potential use could derive from the possibility of identifying other analytical interferences by exploiting the inconsistencies between serum indices and measurement of bilirubin or triglycerides. For example, a sample with a high jaundice index, but with normal bilirubin should alert the operator.

In our case the increased value of lipemic index allowed us to detect the extremely high concentration of cholesterol as a possible explanation for the abnormal peak observed at electrophoresis. In the literature is reported that a cathodic shoulder on the albumin peak can be found with massive hyperlipidaemia (*4*). To the best of our knowledge there are no other papers reporting an association between high cholesterol concentrations and an unusual and distinct anodal peak with respect to the albumin fraction on CZE electropherogram.

Consequently when atypical patterns are present would also be useful to evaluate the lipemic index and or lipemic values. Obviously, there are aspects relating to the use of SI that should be developed. There is still no harmonization between the different analytical platforms on the cut-offs that identify the threshold beyond which interference occurs. Furthermore, there is a clear need to consider serum indices in the same way as any other laboratory parameter and define a quality control assurance system that meets the specific requirements, as required by accreditation regulations (*10*). In conclusion, SI can make a major contribution to improve the performance of the clinical laboratory.
